# Liquid biopsy for minimal residual disease detection in leukemia using a portable blast cell biochip

**DOI:** 10.1038/s41698-019-0102-5

**Published:** 2019-12-02

**Authors:** Bee Luan Khoo, Menglin Shang, Chin Hin Ng, Chwee Teck Lim, Wee Joo Chng, Jongyoon Han

**Affiliations:** 1 Department of Biomedical Engineering, City University of Hong Kong, Hong Kong, China; 2 Critical Analytics for Manufacturing Personalized-Medicine (CAMP) IRG, Singapore-MIT Alliance for Research and Technology (SMART) Centre, Singapore, Singapore; 3 Department of Biomedical Engineering, National University of Singapore, Singapore, Singapore; 4 Department of Hematology-Oncology, National University Cancer Institute, National University Hospital, Singapore, Singapore; 5 Mechanobiology Institute, National University of Singapore, Singapore, Singapore; 6 Department of Mechanical Engineering, National University of Singapore, Singapore, Singapore; 7 Institute for Health Innovation and Technology (iHealthtech), National University of Singapore, Singapore, Singapore; 8 Cancer Science Institute of Singapore, National University of Singapore, Singapore, Singapore; 9 Department of Electrical Engineering and Computer Science, Department of Biological Engineering, Massachusetts Institute of Technology, Cambridge, MA USA

**Keywords:** Diagnosis, Prognosis, Biotechnology, Cancer screening

## Abstract

Long-term management for leukemia is challenging due to the painful and invasive procedure of bone marrow (BM) biopsy. At present, non-invasive liquid (blood) biopsy is not utilized for leukemia, due to lower counts of leukemia blast cells in the blood. Here, we described a robust system for the simultaneous detection and enrichment of rare blast cells. Enrichment of blast cells was achieved from blood with a one-step microfluidic blast cell biochip (BCB) sorting system, without specific targeting of proteins by antibodies. Non-target cells encountered a differential net force as compared to stiffer blast cells and were removed. The efficiency of the BCB promotes high detection sensitivity (1 in 10^6^ cells) even from patients with minimal residual disease. The procedure was validated using actual blast cells from patients with various types of leukemia. Outcomes were compared to current evaluation standards, such as flow cytometry, using BM aspirates. Blast cell detection efficiency was higher in 55.6% of the patients using the BCB as compared to flow cytometry, despite the lower concentrations of blast cells in liquid biopsy. These studies promote early-stage detection and routine monitoring for minimal residual disease in patients.

## Introduction

Bone marrow aspirate (BMA) is a complex mixture of aspirated BM cells, small tissue fragments, and peripheral blood. These BM cells comprise hematopoietic stem cells, mesenchymal stem cells,^1^ fibroblasts, immune cells, and other stroma cell types.^2^ Blasts are immature white blood cells (WBCs) that are normally present in blood and BM at low proportions (<5%). In the management of leukemia, early detection and intervention is the key to promote therapeutic success. The gold standard is BM biopsy,^3^ a procedure that is undesirable for several reasons: (1) high costs, (2) complexity of the surgical procedure, (3) discomfort from the invasive procedure, and (4) increased risk of mortality. Due to these factors, monitoring mutations or blast cell levels from BM biopsies is a tedious process as these procedures must be carried out on a routine basis. In cases whereby such procedures cannot be done (e.g., patient is too weak for surgery), the lack of conclusive screens may affect the evaluation of disease and treatment outcome. Clinicians are keen to introduce rapid, less invasive, and efficient screens for leukemia by employing the use of microfluidic-based assays, which is operated with minimal reagents and samples.^4^

To date, liquid biopsy (blood sampling) has not been fully validated for routine clinical use for leukemia detection. One major reason is the relatively low counts of leukemia blast cells in blood as compared to BM aspirates. Currently, there are no robust procedures to enrich blast cells from the blood. There are few reports on leukemia blast cell enrichment, most of which are not well characterized in terms of purity and efficiency. One example is the centrifugation method based on Ficoll-Paque density gradient,^5^ while the other involves a complex process that focuses on the negative selection of non-blast cells.^6^ In terms of direct detection methods of blast cells, flow cytometry is one of the key procedures utilized. Deep sequencing analysis confirmed the ineffectiveness of flow cytometry on detecting BM blast cells. If applied to blood-derived blast cells, flow cytometry will lead to false negatives as the proportion of blast cells in blood is relatively lower than that in BM and diluted among other blood cell populations (>5%). Patients with minimal residual disease (MRD) have 1 cancer cell in 10,000 or 100,000 leukocytes, while those with chronic stages of leukemia may present even lower levels of blast cells (<5%). Low residual disease levels (MRD <10^−5^) are often not detectable by existing diagnostic procedures. The need for better techniques to detect blast cells at a higher sensitivity is required to improve leukemia diagnosis, especially to explore possibilities of using a minimally invasive approach such as liquid biopsy to replace BM for routine disease monitoring.

We have previously demonstrated the use of inertial microfluidics for sorting circulating tumor cells from peripheral blood of patients with solid tumors,^7,8^ as well as infected malaria blood cells with relevance in disease detection.^9^ In contrast to other cell sorting microfluidics,^10–12^ inertial microfluidics enable high processing rates. Microfluidic cell sorting is hindered by two common limitations: (1) the generation of large output volumes due to the need for high dilution factors and (2) the slow processing speed due to compact cellular interactions that leads to biofouling (clogging) of the device. Subsequent steps to concentrate outputs lead to high degrees of target cell losses, further compromising the sensitivity of target cell detection.

In this research, we described a robust biochip for the simultaneous detection and enrichment of rare blast cells, using non-invasive liquid biopsies from patients. The biochip was validated with samples of patients with acute lymphoblastic leukemia (ALL), myelodysplastic syndrome (MDS), acute myeloid leukemia (AML), acute monocytic leukemia (AMoL), and acute myelomonocytic leukemia (AMMoL). The blast cell biochip (BCB) microfluidic system allowed a label-free and one-step continuous removal of waste and non-target cells to generate concentrated samples of targeted blast cells without the need for specific targeting of proteins by antibodies. Due to this aspect, our procedure allowed detection of a wide variety of blast cells, including lymphoblasts and myeloblasts, to a high degree of sensitivity as compared to current antibody-based evaluation methods such as flow cytometry. The setup could be further incorporated into a continuous flow system under a feedback loop for the concentration of target cells from high initial sample volumes. We believe that these studies will allow the reclassification of blast cell detection limits for early-stage leukemia detection and promote the routine monitoring of MRD in leukemia patients.

## Results

### Design and fabrication of the BCB

**Fig. 1:**
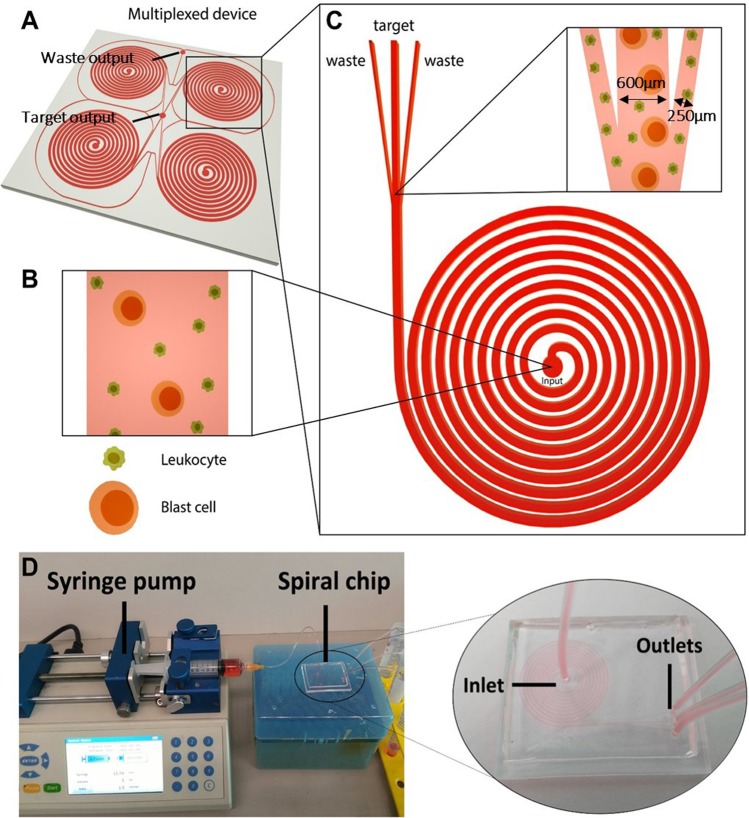
Overview of blast cell isolation using the BCB. **a** 3D overview of the multiplexed BCB for high-throughput processing. Four inputs were located in the middle of each sorting unit. Target and waste outputs were indicated in the figure. **b** Close-up view of the unsorted sample distribution at the input position. **c** Close-up view of the sorted sample distribution at the bifurcation point. **d** Actual biochip incorporated into the system for sorting. Target blast cells were collected in the middle output channel

We designed a microfluidic biochip for the detection of rare counts of leukemia blast cells, based on the principle of inertial focusing. Each biochip could be potentially multiplexed for high-throughput processing (Fig. 1a). In inertial microfluidics, cells within a non-linear microchannel are subjected to two core forces: the net inertial lift force and the dean drag force.^7^ A balance between these forces allows cells of different particle sizes, namely the leukocytes and blast cells (Fig. 1b), to focus at respective positions along the microchannel. Focused streams of cells were separated by a strategically placed bifurcation point (Fig. 1c), allowing the separation of cells undergoing differential net forces. Here, non-target cells, namely the red blood cells (RBCs) and healthy WBCs, encountered a differential net force as compared to the stiffer blast cells of higher nuclear-to-cytoplasmic (N/C) ratio, leading to differential sorting into the waste outlet. Each biochip had the following components: (1) one inlet reservoir, (2) three output reservoirs, and (3) curved microchannels for particle focusing and mixing (Fig. 1d), with size separation thresholds ~138 and 200 μm^2^ for the second and third outermost outlets, respectively (Supplementary Fig. 1). The channel width was 600 µm, and the bifurcation points were 250 µm from the inner wall. The patterned biochip was sealed with another layer of polydimethylsiloxane (PDMS) to allow fluid flow. Samples were introduced through the inlet using a syringe pump, and sorted cells were collected at the three outlets, respectively, depending on their cell size and deformability.

### Characterization of the BCB for blast cell enrichment

A critical factor for the optimization of microfluidic sorting systems is to observe the distribution and sorting efficacy of cell lines during processing. It is pivotal to characterize the device directly with cell samples instead of microparticles due to the difference in the hydrodynamic behavior that deformable cells experience. To mimic the capture of actual clinical blast cell targets in this study, we processed saline buffer samples spiked with various leukemia cell lines. Cell lines provide relevant alternatives for the optimization of device operations and characterization of device efficacy since the primary blast cells from patient samples are relatively fragile, and prolonged processing and repeated handling for labeling may lead to cell loss or damage. Cell lines screened were MOLT-4, HL-60, and KU812E, which corresponded to lymphoblasts, promyeloblasts, and myeloblasts, respectively.

**Fig. 2:**
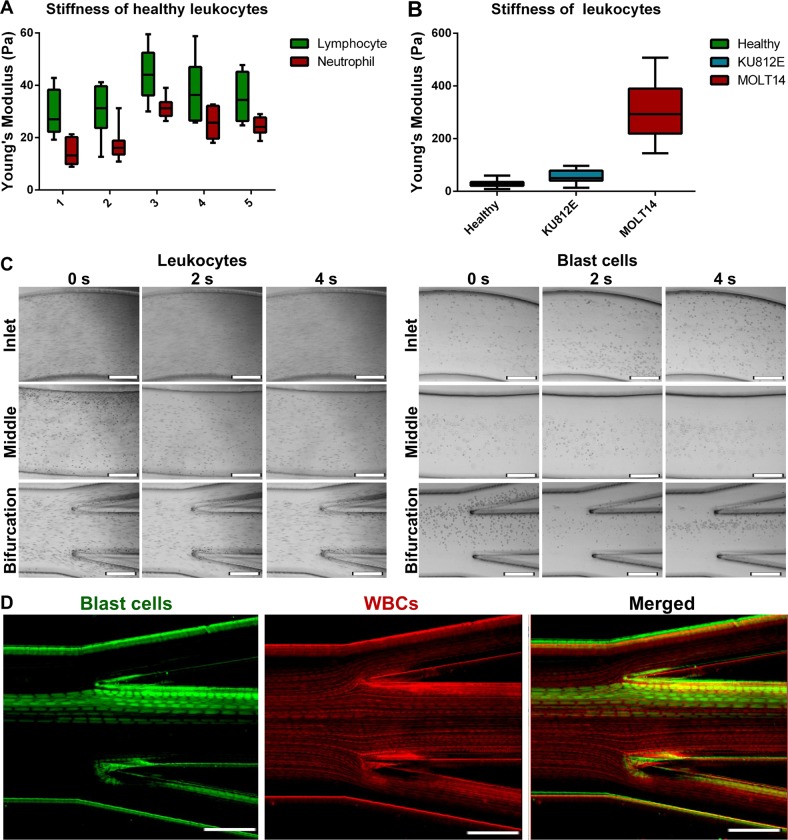
Procedure of enrichment for leukemic blast cells with the BCB. **a** Micropipette aspiration with healthy leukocytes for cell stiffness measurements from five independent screens. **b** Cell stiffness of blast cell types as compared to healthy leukocytes, as determined by their deformability ratio obtained with micropipette aspiration analysis. **c** Characterization of healthy leukocyte and target blast cell distribution under optimal flow rate. Scale bar is 200 µm. Cells were mixed when introduced through the inlet, and the targeted blast cells were gradually focused at the bifurcation point, leaving from the middle output reservoir. **d** Frames captured with a high-speed camera demonstrated the focused streams of target cells among the other blood cells. Target blast cells were shown as green dots and were focused on the middle output. Scale bar is 200 μm

Cells from each cell type have a different range of deformability, as quantified by micropipette aspiration (MPA). Specifically, healthy lymphocytes were slightly stiffer than healthy neutrophils (Fig. 2a), but both studied subsets of healthy leukocytes were significantly more deformable than blast cells (Fig. 2b). MOLT-4 lymphoblasts were stiffer as compared to KU2018 myeloblasts. The increased stiffness of blast cells as compared to their healthy counterparts was comparable to that of other inflammatory conditions reported in the literature, which stated that lymphocyte stiffness significantly increased in chronic lymphocytic leukemia patients as compared to their healthy counterparts.^13^ It had also been shown that neutrophil deformability decreases in sepsis patients.^14^ Such observations could result from the activated state in diseased cells^15–18^ or the high proportion of cell cytoplasm occupied by the nucleus (high N/C ratio) characteristic of blast cells. In terms of activated state, the stiffening effect could be attributed to *N*-formylmethionine-leucyl-phenylalanine (fMLP) activation. fMLP is a chemotactic peptide involved in the immune response of the body, and induction of fMLP results in the activation of a series of downstream signaling pathways that eventually lead to the activation of protein kinase C and increased calcium.^19^ This would eventually cause an increase in actin filament release as well as its polymerization, and, therefore, an increase in cell stiffness.

For the characterization of optimal flow speed, we used leukocyte samples spiked with the stiffest blast cell type (i.e., MOLT-4) to observe cell distributions at the inlet, middle, and bifurcation points with a high-speed camera connected to a phase-contrast light source. Under optimal conditions, the smaller healthy WBCs should migrate away from the perimeters of the outer walls, and then back to the region nearer to the outer walls. Target blast cells, being less deformable and larger, would migrate away from the outer walls and remain focused at the inner portions of the channel. Characterization of target blast cell and WBC distribution under various flow rates suggested that a range of 1.3–1.5 mL/min was suitable for isolating the target cells, but separation at 1.5 mL/min was most efficient, as the focused cell stream was furthest away from the bifurcation point. Hence, we fixed subsequent runs at a flow rate of 1.5 mL/min for consistency (Fig. 2c, Supplementary Fig. 2). Higher flow rates within the optimal range could also increase the isolation of non-target WBC, which would reduce sample purity (Fig. 2d).

**Fig. 3:**
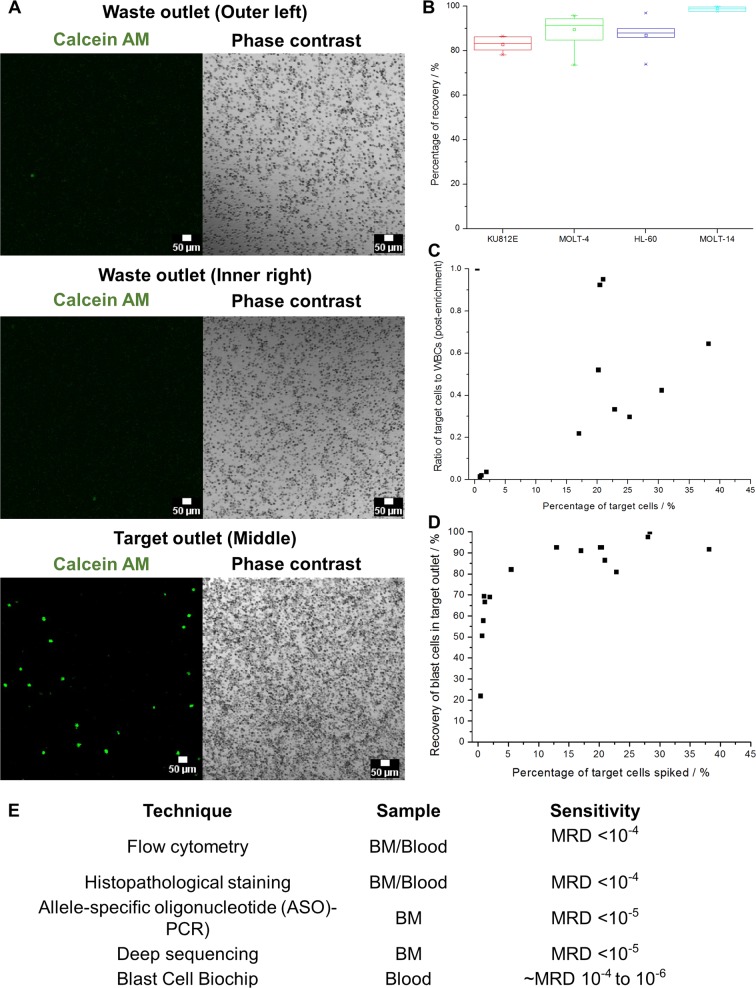
Characterization to validate system robustness and blast cell enrichment efficiency. **a** Visualization of blast cell enrichment from leukocytes with the BCB. Representative confocal images of sorted cell samples from target and waste outlets were presented. Blast target cells were pre-stained with Calcein AM (green). Scale bar is 50 µm. **b** Box plot demonstrating a consistent recovery rate of blast cells of around 89.8 ± 4.4% across three blast cell line types. **c** Scatter plot demonstrating variation in the purity of target cells post-enrichment across samples spiked with different target cell concentrations. The purity value of enriched blast cells (determined by the ratio of target blast cells to other background cells) increased exponentially with the frequency of target cells in the sample (0.1 to 0.65 for samples with >5% blast cells). **d** Scatter plot demonstrating variation in target cell recovery percentages across samples with different target cell concentrations. The recovery rate of samples with low target cell concentration could still be retained around 68.5 ± 1.4%. **e** Comparison of sensitivity for the detection of blast cells in clinical patients with the BCB relative to conventional methods. Logarithmic distribution of recovery efficiency against target cell concentration suggested that the blast biochip system can allow detection even measurable residual disease (MRD) of down to one blast cell per million cells

The morphology of samples remained relatively constant after sorting (Supplementary Fig. 3). The key challenge in separating blast cells from other healthy WBCs is the similarity of cell size (Supplementary Fig. 4). This overlap in physical parameter renders other size sorting-based techniques, such as filtration, impossible. However, the BCB was able to capitalize on the differences in deformability of stiffer blast cells and amplify their differences in migration across the channel width. The difference in cell stiffness can be attributed to the relatively higher N/C ratio in most blast cells, with the nucleus constituting most of the cell volume. Under single processing run with Hoechst-stained MOLT-4 cells spiked in healthy blood samples, we could demonstrate a high recovery of blast cells in the middle outlet (Fig. 3a). These samples were spiked with clinically relevant MOLT-4 cell counts, which represented samples with high blast cell counts of >5%. Enriched MOLT-4 samples reflected consistent recovery rates of 89.8 ± 4.4% (Fig. 3b). Similar recovery efficiencies were observed for other leukemia cell lines (HL-60: 87.3 ± 8.3% and KU812E: 86.5 ± 4.6%) spiked in healthy blood samples.

With actual clinical samples from leukemia patients, blast cells were presented at lower frequencies than other blood cells, including nucleated RBCs (0–60%), lymphocytes (0–20%), and monocytes (0–95%) (Table 1). The concentration of cells affected the efficacy of cell sorting and focusing along the channel width, hence a simple step of RBC lysis was implemented before sorting to keep samples within the optimal cell concentration (>1 million cells/mL). The RBC lysis step had been previously validated to exert minimal cellular damage, and cells remained consistent in terms of viability and morphology before and after lysis.^7^

**Table 1:** Cell composition percentage from clinical bone marrow aspirates of patients estimated by flow cytometry

	ID	Type	Promyelocyte	Myelocyte	NRBC	Lymphocyte	Monocyte	Macrophage
1	P-J	ALL	0	6	17–18.2	6–18.3	0–0.4	0
2	GLL	MD	0–2.9	10	44.1–48	0–6.5	0–0.6	4
3	LSK	AML	0	3	8–11.7	13–16.7	0–10.9	3
4	ABB	ND	ND	ND	ND	ND	ND	ND
5	MAS	AML	0	3	46.9–56	2–14.4	0–0.5	0
6	D-D	AML	0	1	0.4–1	1	0–0.1	0
7	NTM	AML	0–1.1	2	8.4–16	7–14.5	0–3.1	0
8	DSP	AML	2	4	11–19.5	12.7–13	6–20.6	2
9	GJDDC	AML	1–1.2	6	2.9-8	0–2.1	11–49.2	0
10	GKL	AMMoL	1–5.1	5	1.8–7	2–7.5	11–55.8	0
11	WYC	AMMoL	2	0	0.9–4	2–5.5	6–47.5	0
12	KBH	MD	3–18.8	20	8.1–21	3–12	1–43.7	0
13	MTSB	AMMoL	2	6	1.7–3	1.9–3	17–36.5	0
14	CPF	AMoL	0	0	2	1	5	1
15	TKH	AMoL	29	2	4	0	12–94.8	0

*NRBC* non-red blood cells, *ID* patient identification code, *ALL* acute lymphoblastic leukemia, *MD* myelodysplastic leukemia, *AML* acute myeloid leukemia, *ND* not determined, *AMMoL* acute myelomonocytic leukemia

To validate the application of the BCB for detecting low counts of blast cells from blood, we first used samples spiked with leukemia cell lines to represent clinical blast cells from the blood of patients with residual or chronic disease (with lower blast cell counts of <5%). Concentration and purity of target cells correlated with recovery efficiency. In this system, the purity of enriched blast cells (determined by the ratio of target blast cells to other background cells) increased exponentially with the frequency of target cells in the sample (0.1–0.65 for samples with >5% blast cells) (Fig. 3c). At low spike counts, the system could still retain a purity ratio of 0.005–0.34 for samples with <5% blast cell counts. Similarly, the recovery rate increased exponentially with target cell concentration, but at low spiked cell counts, the system could still retain an effective recovery rate of 68.5 ± 1.4% (Fig. 3d).

From the recovery rates of samples spiked with blast cells, the efficiency of this system extrapolated to a minimal detection rate of disease in samples of five blast cells among one million leukocytes (MRD 10^−6^) (Fig. 3e). This is important as a sensitive detection rate is pivotal to establishing the threshold of blast cells for detection. The current threshold for AML diagnosis is at 20% blast cells,^20^ and a more sensitive means of blast cell enrichment may allow clinicians to detect cases of MRD after treatment or cases of relapse at lower blast cell count levels.

### Isolation and concentration of rare clinical blast cells from liquid biopsy

After validation of the BCB for blast cell capture with cell lines, we processed blast cells from actual clinical blood samples as a proof of concept. Instead of conventional BM aspirates, we obtained samples from liquid biopsies (2–3 mL). Whole blood from leukemia patients was withdrawn and processed within 6 h to ensure optimal sample conditions. Overall, liquid biopsy samples were obtained from 15 patients (single blood draw) with advanced stages of leukemia.

**Fig. 4:**
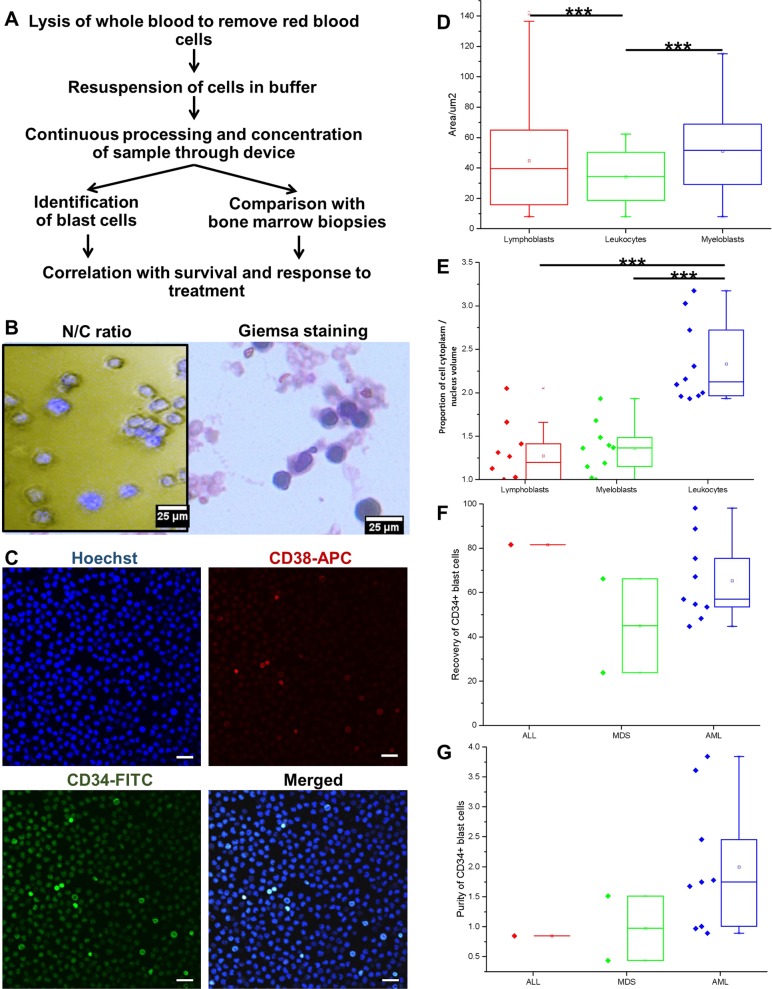
Clinical proof of concept for blast cell capture and enrichment from the liquid biopsy. **a** The workflow of sample processing and analyses for blast cell detection. **b** Downstream analyses to demonstrate the high proportion of cell cytoplasm occupied by the nucleus in enriched blast cells. (Left) Nuclei labeling with Hoechst dye. Scale bar is 25 µm. (Right) Histopathology analysis with Giemsa stains. **c** Immunostaining with antibodies to target CD34^+^CD38^−^ blast cell subpopulations. Scale bar is 50 μm. **d** The average size of clinical blast cells and leukocytes by area. ***P < 0.001. **e** Proportion of cell cytoplasm over nucleus volume for clinical blast cells and leukocytes. ***P < 0.001. **f** Recovery rates of CD34^+^ clinical blast cells. **g** The purity of CD34^+^ clinical blast cells after enrichment

Further BM aspirate report and flow cytometry analysis from BM samples taken at the same time point confirmed their diagnosis, which ranged from ALL, MDS to various subtypes of AML (Table 1). Other demographics associated with the patient cohort were listed in Supplementary Table 1. Among these, information on the longitudinal cohort study was obtained, including tumor response, patient relapse, and patient survival statistics. Blood samples were briefly processed to remove RBCs using an RBC lysis procedure (Fig. 4a), which had been previously demonstrated to incur minimal cell damage and loss of nucleated cell fraction after lysis.^21^ Nine of the clinical samples (*n* = 15) were processed and analyzed to identify the proportion of cells in the target outlet as well as the percentage of patient-derived CD34^+^CD38^−^ blast cells using immunostaining. The remaining clinical samples were utilized for the optimization and calibration of device. Results from the nine samples were correlated with the BM aspirate report and flow cytometry report obtained with BM aspirates from the same patient (Table 2). From the clinical samples, we determined that the blast cells were of high N/C ratio, as verified with various downstream analyses such as nuclei labeling, histopathology staining (e.g., Giemsa), and immunostaining for blast cell markers (Fig. 4b, c), demonstrating the reliability of the system for clinical blast cell detection.

**Table 2:** Blast cell counts of clinical samples from patients using flow cytometry and BCB

ID	Conclusion	BM aspirate: CD34^+^	BM aspirate: other	BM aspirate: total	BM flow report: CD34^+^	BCB: CD34^+^	BCB: target outlet (total)
P-J	ALL	0	49 (lymphoblast)	49	0.2		
GLL	MD	3		3	6.1		
LSK	AML	60		60	42	17.77	48.01
ABB	ND	ND		ND	ND		
MAS	AML	24		24	28.3	58.83*	14.24
D-D	AML	42	54 (monoblast)	96	0.1	1.52*	32.67
NTM	AML	32	40 (proerythroblast)	72	57.3		
DSP	AML	52		52	30.5	9.54	58.61
GJDDC	AML	20	46 (monoblast)	66	0.1	17.96*	37.81
GKL	AMMoL	9	46 (monoblast)	55	2.1	2.42*	83.59
WYC	AMMoL	13	71 (monoblast)	84	24	7.72	77.66
KBH	MD	22	3 (monoblast)	25	5.3	17.56*	43.76
MTSB	AMMoL	10	45 (monoblast)	55	27.5	3.23	36.16
CPF	AMoL	0	85 (monoblast)	85	ND		
TKH	AMoL	0	39 (monoblast)	39	0.1		

Flow cytometry analyses were obtained with BM aspirates of higher blast cell concentrations. BCB analyses were obtained with a liquid biopsy of lower blast cell concentrations. An asterisk (*) denotes the cases where blast cell detection efficiency was higher with the BCB, despite the lower concentrations of blast cells in liquid biopsy

*ID* patient identification code, *ALL* acute lymphoblastic leukemia, *MD* myelodysplastic leukemia, *AML* acute myeloid leukemia, *ND* not determined, *AMMoL* acute myelomonocytic leukemia, *BM* bone marrow, *BCB* blast cell biochip

The purity of enriched target blast cells is pivotal in terms of setting new diagnostic thresholds as well as for downstream analytical purposes. Contaminating materials from a sample can induce noise and prevent rare signals from detection, leading to false negatives or false positives. As previously discussed, the key challenge to enrich blast cells from the blood was due to the overlapping cell size range as compared to other leukocytes (Supplementary Fig. 4). We estimated that the maximum range of cell areas for CD34^+^CD38^−^ blast cells was up to 166.8 μm^2^ (Fig. 4d). Both lymphoblasts and myeloblast subtypes were significantly larger than most leukocytes in clinical samples. The average area of myeloblasts was slightly larger than the average area of lymphoblasts. The proportion of total cell area filled by the nucleus for both blast cell types was higher than that of leukocytes, reflecting the presence of a large nucleus, which is atypical of blast cell phenotype. The proportion of cell area to the nucleus (1 over N/C) in enriched lymphoblasts (1.27 ± 0.35) was slightly lower than that of enriched myeloblasts (1.35 ± 0.29) (Fig. 4e), suggesting that the nucleus of lymphoblasts were larger, albeit the difference was not significant. However, this slight difference could be a result of the overall increase in stiffness of lymphoblasts as compared to myeloblasts (Fig. 2b), which then led to the better recovery of lymphoblasts than myeloblasts in cell lines (Fig. 3b). Most interestingly, this variation in blast cell recovery efficacy was also reflected in clinical samples (Table 2), with AML samples containing lymphoblasts (AML: 65.3 ± 18.6%) having a better recovery rate than MDS samples containing myeloblasts (MDS: 45.0 ± 30.0%; Fig. 4f). The same observation was seen with an ALL sample containing lymphoblasts (ALL: 81.6%), albeit a larger sample size would be required to demonstrate the significance of these trends.

Similarly, the purity ratio of blast cells to leukocytes recovered from AML clinical samples (AML: 2.0 ± 1.1%) was much higher than that of MDS samples (MDS: 1.0 ± 0.6%; Fig. 4g). In the AML clinical samples, we could deplete leukocytes by a range of 1.28–14.4-folds in a single run (Supplementary Fig. 5), leading to a much purer blast cell fraction at the collection outlet. The depletion value was a function of multiple factors, including the concentration of target cells, quality of blood sample, size of abnormal cells, and so on. Here, we demonstrated an exponential relationship between the concentration of target cells and depletion ratio.

It is important to note that blast cells are heterogeneous and may comprise of populations that are not CD34^+^CD38^−^.^22,23^ Indeed, besides CD34^+^CD38^−^ blast cells, we also observed CD34^−^ cells with high N/C ratio in target outlets (Supplementary Fig. 6a). These could be CD34^−^ blast cells or leukemia stem cells, which were associated with worsened prognosis and sensitivity to therapy.^24^ Other megakaryocyte-like large cells were also captured (Supplementary Fig 6b), and their presence was associated with acute megakaryoblastic leukemia.^25^ The enrichment of CD34^−^ blast cells and megakaryocyte-like cells using the BCB highlight the capability of this technique to identify other leukemia subtypes potentially, and can be subsequently utilized for correlation to patient overall survival.

### Simultaneous isolation and concentration of target blast cells

Using a single biochip, we could recover blast cells at a sensitivity higher than that demonstrated by current evaluation methods (Fig. 3e). However, cell concentration at the outlets was still relatively low due to a large output volume, which was not desirable for downstream analyses such as deep sequencing. Although a simple centrifugation step might suffice to remove residual fluid output, we attempted to further increase the purity of target cells by connecting the sorted sample back into the inlet to form a feedback loop. Similar feedback loops have been previously proposed for other applications.^26^ This step is especially beneficial for samples comprising target cells with a similar cell size range as compared to the background cells, preventing absolute purification with a single run.

**Fig. 5:**
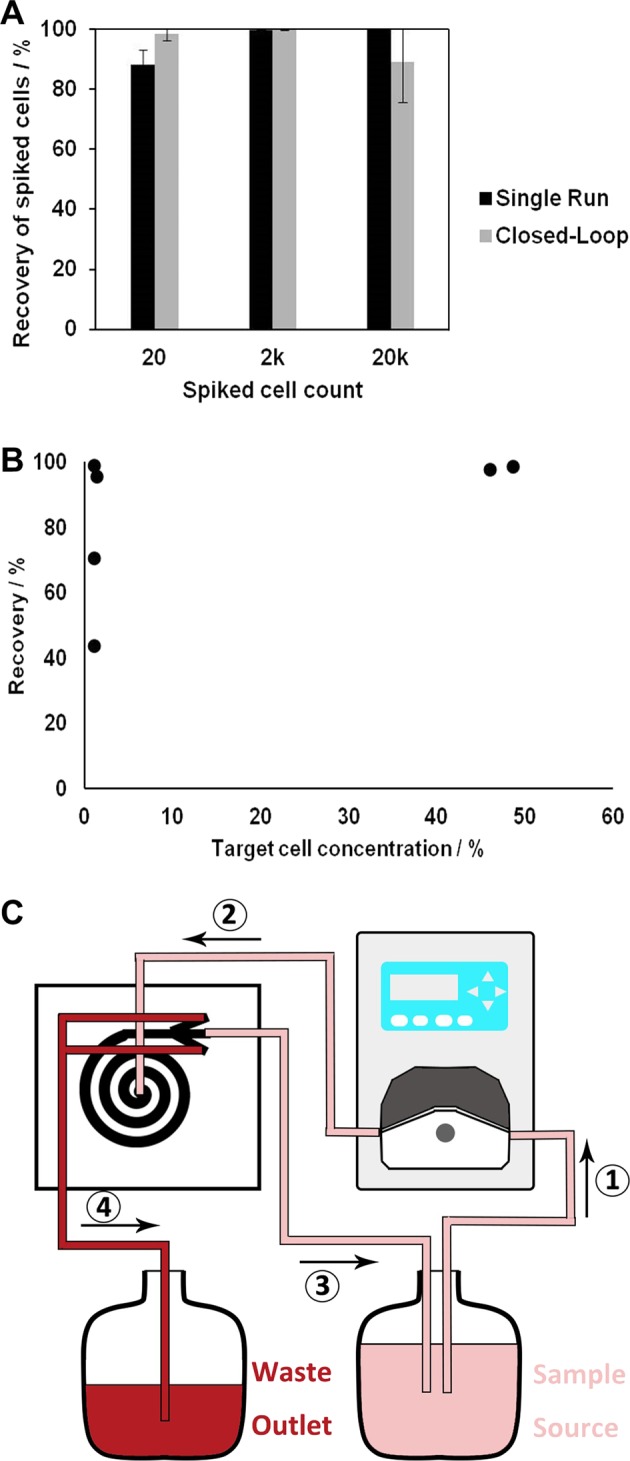
A feedback loop procedure for the simultaneous enrichment and concentration of rare target blast cells. **a** Recovery rates for samples with different concentrations of spiked cell count under continuous processing with the feedback loop. Spiked cell count refers to the number of blast cells spiked into 1 mL of blood sample. **b** The recovery rate of low target blast cell counts at different target cell concentration. **c** Schematics of the system after incorporation of a feedback loop. A peristaltic pump could be used to sort the sample repeatedly. By recycling blast cells output back to the sample source, we could continuously remove non-target cell populations such as the WBCs, allowing higher purity of target cells recovered

The feedback loop to the sample source allowed the continuous removal of smaller particles, which cannot form a focused stream within the microchannel, as well as fluid output through waste disposal. Several methods were attempted to achieve this feedback processing. First, we tried to process the sample output from the target outlet repeatedly and observed an exponential increase in target cell purity with repeated runs. With this method of the feedback loop, we were able to maintain high recovery rates of blast cells (96 ± 5.65%), even using samples spiked with low counts of blast cells (20 cells; Fig. 5a). As with single runs, recovery rate increased exponentially with target cell concentration. Although recovery under a feedback loop system dropped at low spike counts (<1.1%), a recovery rate of at least 40% could still be retained (Fig. 5b).

Feedback could also be enabled through continuous flow using a peristaltic pump (Fig. 5c). Due to the pulsatile effects of the peristaltic pump, the flow rate here varied during continuous processing, and an amount of cell loss was inevitably sustained with each run.

Overall, single runs with the BCB were sufficient to concentrate blast cells from AML samples of >10% target cell concentration. The ability to detect low blast cell counts rapidly opens up new possibilities for leukemia detection. The BCB can also be coupled with existing techniques such as deep sequencing to achieve new thresholds of blast cell detection for early-stage disease, MRD, or relapse. A manual feedback loop procedure is recommended for samples of low target cell concentration, especially if a high concentration of target cells is required for downstream molecular analyses.

## Discussion

**Fig. 6:**
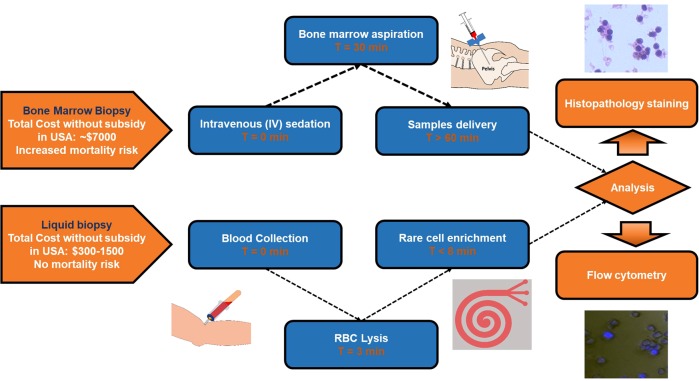
Schematic overview of the procedure of leukemia detection via conventional BM biopsy and liquid biopsy. In conventional methods, blast cells are extracted from BM via an invasive and painful surgical procedure. Detection threshold limits affect the effective identification of disease in patients with low blast cell counts. Conversely, blast cells can be isolated rapidly (1.5 mL/min) from liquid biopsies using a continuous flow microfluidics assay. In this procedure, a small volume of blood was obtained from patients for blast cell enrichment with microfluidics. A liquid biopsy is a relatively non-invasive procedure and is cost-effective, promoting routine evaluation for disease monitoring

The motivation to work with liquid biopsies instead of BM aspirates is to allow convenient detection of blast cells without incurring BM aspiration (Fig. 6). BM aspiration is the conventional method used for detection, whereby blast cells are extracted from BM via an invasive procedure. Cells may be identified by histopathological analysis or flow cytometry. Besides, BM biopsies are painful, costly procedures and may increase mortality risks. Detection threshold limits also affect the effective identification of disease in patients with low blast cell counts (<5%). Furthermore, liquid biopsy sampling allows wider applicability to cases where the patient cannot handle routine (every few weeks) BM screens. In all, 13.3% (*n* = 15) of patients had no corresponding data (Table 2) from BM analyses due to similar reasons.

Here, we demonstrate the rapid enrichment of blast cells from liquid biopsies using a continuous flow BCB system (Fig. 1). Various types of cells can be used for the diagnosis of leukemia subtypes. In the case of AML, an increase of blast cell populations serves as one of the key determinants for diagnosis. The morphology of these cells is subsequently evaluated by histopathological or immunohistochemistry analysis to validate the specific subtypes as AML is genetically diverse. In this study, AML served as our key focus for analysis (78.6%, *n* = 15). Currently, there is no standard approach to detect the presence of leukemic cells, which reflect relapse risk. We aim to utilize the BCB to extract these leukemic blast cells without the need for genomic assays, based on differential cell size and stiffness of the blast cells as compared to other blood components. The BCB can serve as a complementary tool to histopathological or immunohistochemistry analysis, by concentrating and isolating blast cell fractions. This procedure will allow the detection of low blast cell counts and facilitate detection of MRD, of which blast cell counts remained at relatively low levels (1 cancer cell in 10,000 or 100,000 leukocytes). Patients with chronic stages of leukemia may be presented with even lower levels of blast cells (<5%). These low residual disease levels (MRD <10^−5^) are often not detectable by existing diagnostic procedures. The concentration of blast cells for histopathological or immunohistochemistry analysis can also improve the accuracy of diagnosis by facilitating detection of leukemic cells for phenotyping or subsequent genomic profiling.

In this procedure, the high recovery and concentration factor obtained provided an opportunity to isolate blast cells from the very small volume of blood, such as a drop of blood from a fingertip, before downstream analyses. A sensitive detection rate is pivotal to re-establish the threshold of blast cells for detection. The current threshold for AML diagnosis is set at 20% blast cells, which is relatively high considering that normal levels of blast cells were usually around <5%.^20^ Such a coupling of complementary techniques will drastically reduce the blast cell detection limit and allow clinicians to detect cases of MRD after treatment or cases of relapse.

Leukemia blast cells (13–16 µm) share a similar cell size range with its background cells (8–20 µm) (Supplementary Fig 4). Enrichment of blast cells from other leukocytes is usually challenging due to overlapping size range and varied morphology. The BCB capitalized on the distinct differences in deformability to remove more deformable cells. The deformability of cells has been previously reported to affect the sorting efficiency of inertial microfluidic devices.^27^ Although the focusing position of cells in inertial microfluidics does not depend on cell shape, cells’ hydraulic diameter, which is affected by deformability plays an important role.^28^ The more deformable cells will tend to move away from the channel wall as compared to the less deformable cells of the same size. Therefore, the deformability of cells has an impact on the focusing position during sorting.^27^

Blast cell counts in blood are known to be considerably lower than BM aspirates.^29^ Despite that, the resultant percentage of CD34^+^CD38^−^ blast cells recovered with the BCB was still higher in 55.6% of the samples screened (*n* = 15), as compared to parallel results obtained from standard diagnostic methods such as flow cytometry using BM samples (Table 2). Hence, the BCB was able to allow robust detection of blast cells from blood, even at a higher sensitivity than gold standards using BM aspirates. Such findings may be pivotal in the development of a new benchmark to replace BM aspirates as the core source of disease detection.

As compared to liquid biopsy, BM aspirates also comprise small tissue fragments. Depending on the size of the tissue, BM aspirate samples may have to undergo a pre-filtration step to remove larger tissue aggregates before processing with the BCB. Larger tissue aggregates can affect cell sorting within the channel or obstruct flow completely (biofouling). Smaller tissue fragments may be easily trapped at the inlets of the BCB by introducing slight modifications to trap larger particles before they enter the microfluidic channel.

The integration of a manual feedback loop may allow the continuous removal of non-target particles to generate simultaneous concentration and purification of rare target cells and can be highly effective for samples that cannot be purified (Fig. 5). Enriched cancer samples will be utilized for downstream analysis, such as polymerase chain reactions, sequencing, immunostaining, and fluorescence in situ hybridization. Alternatively, such systems can be applied to the enrichment of small particles such as bacteria, protein, or genetic material, to be purified with the removal of larger cells. A portable and low-cost continuous flow microfluidics separation system with a pulse-free system can also be developed for cell-based diagnostics in countries without proper biological equipment.

Overall, the ability to detect leukemia with non-invasive and inexpensive techniques provides a powerful tool for clinicians to monitor residual disease and to enable early detection. The simplicity of system setup allows potential multiplexing, promoting the concentration of large sample volumes, such as blood, urine, or diluted biological samples. Removal of background cells leads to high sensitivity of target cell detection (~MRD 10^−6^, which is more sensitive than current techniques), allowing our system to be suitable for early-stage diagnosis, detection of MRD, or chronic stages of leukemia. Heightened blast cell counts in the peripheral blood may reflect worsened prognosis. Therefore, we hope that the sensitivity of our system will allow clinicians to revisit the current thresholds of blast cells in the blood and promote early detection of leukemia for prompt intervention.

## Methods

### Fabrication of the BCB

The microfluidic chips were fabricated using standard soft-lithography techniques in PDMS described elsewhere.^7^ After the fabrication of individual biochip layers, the sealed biochip was obtained by manual alignment and oxygen plasma bonding. The respective fluidic inlets and outlets were punched into the layers prior bonding.

### Cell culture

Three human leukemia blast cell lines (MOLT-4, HL-60, and KU812E) were used in this study. MOLT-4 (ATCC, CRL-1582) are lymphoblasts from adult and childhood lymphoblastic leukemia. HL-60 (ATCC, CCL-240) are promyeloblasts. KU812E (ATCC, CRL-2099) contained myeloblasts from patients with chronic myelogenous leukemia. All the cell lines were maintained in a T25 flask with Roswell Park Memorial Institute medium (RPMI) (Life Technologies, 11875-119) along with 1% of penicillin–streptomycin and 10% of fetal bovine serum (Thermo Fisher, Singapore) at 37 °C and 5% CO_2_. The cell media were changed every 2–3 days, and cells were harvested when their confluency reached 80%.

### Cell harvesting

The media were aspirated from the cell culture flask, and 1 mL of 1× phosphate-buffered saline (PBS) was added (Vivantis Inc., Cat. #PB0344-1L). The flask was gently shaken to remove debris and dead cells, and the PBS was aspirated. One milliliter of trypsin (Gibco, Ref. #25300–054) was added to the cell flask, which was placed into the incubator at 37 °C and 5% CO_2_ for 5 min. Afterwards, 1 mL of fresh media was added to terminate the reaction. The contents of the cell culture flask were transferred to a Falcon tube (Corning, Ref. #352096) and centrifuged for 3 min at 1200 r.p.m. (Beckman Coulter, Allegra X-15R). The supernatant was aspirated, and cells were resuspended in 1 mL PBS.

### Deformability measurements using MPA

For healthy neutrophil and lymphocyte isolation, lymphocytes were isolated using EasySep^TM^ Direct Human Total Lymphocyte Isolation Kit (StemCell Technologies, Canada) following the manufacturer’s protocol. Neutrophils were isolated using EasySep^TM^ Direct Human Total Neutrophil Isolation Kit following the manufacturer’s protocol. These isolation kits used magnetic beads to select intended cells in the blood sample negatively. For blast cell measurements, cell lines were centrifuged at 300 × *g* RCF (relative centrifugal force) for 3 min. The cell pellet was resuspended in media to achieve 5k cells/mL concentration. Over 20 cells were studied for each experimental group. The isolated subtypes were kept on the ice and immediately processed with our microfluidic biochip, followed by MPA. According to previous studies,^30^ a cell can be modeled as a homogeneous elastic solid, and its elastic modulus can be obtained from the following formula (1):

1 $${\mathrm{1:}}\;\Delta {P} = {G}\frac{{2\pi L_{\mathrm{p}}}}{{3R_{\mathrm{p}}\emptyset _{\mathrm{p}}}},$$

where Δ*P* is aspiration pressure, *E* is Young’s elastic modulus, ∅_P_ is wall function which is 2.1, *L*_P_ is aspiration length of the cell into the pipette, and *R*_p_ is the radius of the aspiration pipette. To obtain cell Young’s modulus using MPA, the cells were aspirated into a micro-sized pipette where the aspiration pressure increased from 0 to 600 Pa with a constant rate of 3.27 Pa/s. Cell deformation was recorded at 1 frame per second. Frames with observable cell protrusion were selected for analysis. Manual measurement of aspiration length was done using the ImageJ software, and slope of the fitted line was used to calculate elastic modulus of each cell.

### Lysis of whole blood for processing

This study was approved by respective institutional review boards and the local ethics committee (National Healthcare Group) (DSRB Reference 2016/00770). Informed and written consent was obtained from all patients. Fifteen blood samples from patients with advanced leukemia were collected. Since AMMoL and AMoL are considered subtypes of AML, their results are classified together with those from other AML samples in this study. RBC lysis was done to obtain nucleated cells for processing. Blood was mixed with RBC lysis buffer (1:3 ratio; Life Technologies, Carlsbad, CA) under gentle agitation for a maximum of 3 min, and centrifuged at 1000 × *g* for 5 min to concentrate the intact nucleated cells. The supernatant containing lysed RBC debris and plasma was decanted, and the resultant cell pellet was immediately washed once with PBS.

### Preparation of blood samples spiked with blast cells

Two hundred microliters of cell suspension and 50 μL of PBS were mixed in an Eppendorf tube. One microliter of CellTracker Green (Life Technologies, Ref. #C2925, Eugene) was pipetted into the tube, which was placed in an icebox for 10 min. The concentration of stained cells was calculated using a hemocytometer (NanoEnTek, Neubauer Improved DHC-N01), and respective counts of blast cells were added. To accurately determine the spiked cell counts, three sets of 100 μL cell stocks were imaged in wells of a 96-well plate. The average counts were obtained to arrive at the initial spiked cell count. The final spiked cell suspension was diluted with PBS to a total volume of 10 mL and treated with 300 μL 0.2% of Poloxamer 188 solution (Sigma-Aldrich, Ref. #P5556) before processing within the system.

### System processing

Before enrichment, the chip was primed by flowing PBS for 60 s at 1.5 mL/min. For single runs, nucleated cell fraction containing WBC and blast cells were resuspended to achieve a concentration of ≤100,000 cells/mL with saline buffer. Samples were processed once through the biochip at a flow rate of 1.5 mL/min. For continuous separation, nucleated cell fraction was resuspended to 10 mL of saline buffer supplemented with Poloxamer 188. Poloxamer 188 was introduced to prevent cells from sticking to the microchannels, promoting cell recovery, and maximizing focusing of cells for sorting. The diluted sample could be manually passed through the BCB for a single or several cycles. Alternatively, a peristaltic pump (SG Biotic, Singapore) was used to flow the cell solution to form a feedback loop procedure, collecting solutions from the target outlet (middle) back into the inlet of the biochip, until the final volume of the inner outlet solution was ~500 μL. If a higher flow rate of the peristaltic pump is required, a multiplexed biochip should be used to allow a split of the sample flow for optimal processing.

### Immunostaining and data analysis

The sample was processed with dyes (Hoechst 33342 dye (1:250, 1M, Sigma)) and primary antibodies (anti-rabbit CD34, anti-mouse CD38; 1∶250, Miltenyi Biotec Asia Pacific, Singapore)) to identify blast cells, and the enumerated blast cell counts were used for correlation with BM aspiration and flow cytometry results. Respective 488 or 594 secondary antibodies (1:250, Abcam, Cambridge, UK) were used with the primary antibodies. Staining was carried out under dark conditions on ice for 30 min. Samples were transferred to a 96-well plate for imaging with an inverted confocal microscope (Olympus Fluoview FV1000, USA) (Emission filters ET460/50m, ET535/50m, and ET 605/70; Olympus, Tokyo, Japan), and cell counting was automated with a custom ImageJ script.

For estimations of cell and nucleus volume, images of Hoechst- and Calcein AM-stained cells were obtained with the selection tool via ImageJ. Proportion of the cell cytoplasm to nucleus volume was calculated, with 1:1 stating that the cytoplasm is of the same volume as the nucleus.

### Histological staining of enriched blast cells

Histopathological morphology of the cultured cells was observed via standard Wright–Giemsa staining procedures at the Department of Hematology, National University of Singapore. Staining was done on frosted slides (Thermo Fisher Scientific) with cell spots fixed in methanol.

### Statistical analysis

All error bars represented the standard deviation of triplicate samples. Groups were compared using the Student’s *t* test to evaluate associations between independent variables, and the *p* values were obtained. Adjusted multivariate analyses for continuous independent variables (to other variables) required larger sample sizes and were not used in this study. Further, Cox regression (investigation of multiple variables) was also not carried out because of the small sample size.

### Reporting summary

Further information on research design is available in the Nature Research Reporting Summary linked to this article.

## Supplementary information

Supplementary information

Reporting summary

## References

[1] Friedenstein AJ (1980). Stromal mechanisms of bone marrow: cloning in vitro and retransplantation in vivo. Haematol. Blood Transfus..

[2] Birbrair A, Frenette PS (2016). Niche heterogeneity in the bone marrow. Ann. NY Acad. Sci..

[3] Frisch B, Bartl R (1986). Bone marrow histology in myelodysplastic syndromes. Scand. J. Haematol. Suppl..

[4] Whitesides GM (2006). The origins and the future of microfluidics. Nature.

[5] Tong XZ (2012). Apatinib (YN968D1) enhances the efficacy of conventional chemotherapeutical drugs in side population cells and ABCB1-overexpressing leukemia cells. Biochem. Pharm..

[6] Kornblau SM, Coombes KR (2011). Use of reverse phase protein microarrays to study protein expression in leukemia: technical and methodological lessons learned. Methods Mol. Biol..

[7] Warkiani ME (2016). Ultra-fast, label-free isolation of circulating tumor cells from blood using spiral microfluidics. Nat. Protoc..

[8] Warkiani ME (2014). Slanted spiral microfluidics for the ultra-fast, label-free isolation of circulating tumor cells. Lab Chip.

[9] Warkiani ME (2015). Malaria detection using inertial microfluidics. Lab Chip.

[10] Nima ZA (2014). Circulating tumor cell identification by functionalized silver-gold nanorods with multicolor, super-enhanced SERS and photothermal resonances. Sci. Rep..

[11] Nagrath S (2007). Isolation of rare circulating tumour cells in cancer patients by microchip technology. Nature.

[12] Jing T (2015). Jetting microfluidics with size-sorting capability for single-cell protease detection. Biosens. Bioelectron..

[13] Zheng, Y. et al. Decreased deformability of lymphocytes in chronic lymphocytic leukemia. *Scientific Rep.* **5**, 7613 (2015). 10.1038/srep07613. 10.1038/srep07613 PMC4287721 25573422

[14] Kovach MA, Standiford TJ (2012). The function of neutrophils in sepsis. Curr. Opin. Infect. Dis..

[15] Hu M, Wang J, Zhao H, Dong S, Cai J (2009). Nanostructure and nanomechanics analysis of lymphocyte using AFM: from resting, activated to apoptosis. J. Biomech..

[16] Suemori T (2009). Impairment of leukocyte deformability in patients undergoing esophagectomy. Clin. Hemorheol. Microcirc..

[17] Dadgostar H (2006). Hemorheologic abnormalities associated with HIV infection: in vivo assessment of retinal microvascular blood flow. Invest. Ophthalmol. Vis. Sci..

[18] Nishino M (2005). Serial changes in leukocyte deformability and whole blood rheology in patients with sepsis or trauma. J. Trauma Acute Care Surg..

[19] Entschladen F, Gunzer M, Scheuffele CM, Niggemann B, Zänker KS (2000). T lymphocytes and neutrophil granulocytes differ in regulatory signaling and migratory dynamics with regard to spontaneous locomotion and chemotaxis. Cell. Immunol..

[20] Ferrara F, Schiffer CA (2013). Acute myeloid leukaemia in adults. Lancet.

[21] Khoo BL (2014). Clinical validation of an ultra high-throughput spiral microfluidics for the detection and enrichment of viable circulating tumor cells. PLoS ONE.

[22] Levine JH (2015). Data-driven phenotypic dissection of AML reveals progenitor-like cells that correlate with prognosis. Cell.

[23] Bonnet D, Dick JE (1997). Human acute myeloid leukemia is organized as a hierarchy that originates from a primitive hematopoietic cell. Nat. Med..

[24] Zeijlemaker W (2016). A simple one-tube assay for immunophenotypical quantification of leukemic stem cells in acute myeloid leukemia. Leukemia.

[25] Dohner H (2010). Diagnosis and management of acute myeloid leukemia in adults: recommendations from an international expert panel, on behalf of the European LeukemiaNet. Blood.

[26] Choi K (2018). Negative selection by spiral inertial microfluidics improves viral recovery and sequencing from blood. Anal. Chem..

[27] Hur SC, Henderson-MacLennan NK, McCabe ER, Di Carlo D (2011). Deformability-based cell classification and enrichment using inertial microfluidics. Lab Chip.

[28] Hur SC, Choi S-E, Kwon S, Carlo DD (2011). Inertial focusing of non-spherical microparticles. Appl. Phys. Lett..

[29] Amin HM (2005). Having a higher blast percentage in circulation than bone marrow: clinical implications in myelodysplastic syndrome and acute lymphoid and myeloid leukemias. Leukemia.

[30] Theret DP, Levesque MJ, Sato M, Nerem RM, Wheeler LT (1988). The application of a homogeneous half-space model in the analysis of endothelial cell micropipette measurements. J. Biomech. Eng..

