# Supplementary material for: Liquid biopsy for minimal residual disease detection in leukemia using a portable blast cell biochip
Source: NPJ Precis Oncol. 2019 Dec 2;3:30. doi: 10.1038/s41698-019-0102-5 (PMC6889137; doi:10.1038/s41698-019-0102-5)
Supplement: Supplementary file 1 — Supplementary information [file 41698_2019_102_MOESM1_ESM.docx]

**Supplementary Figures**


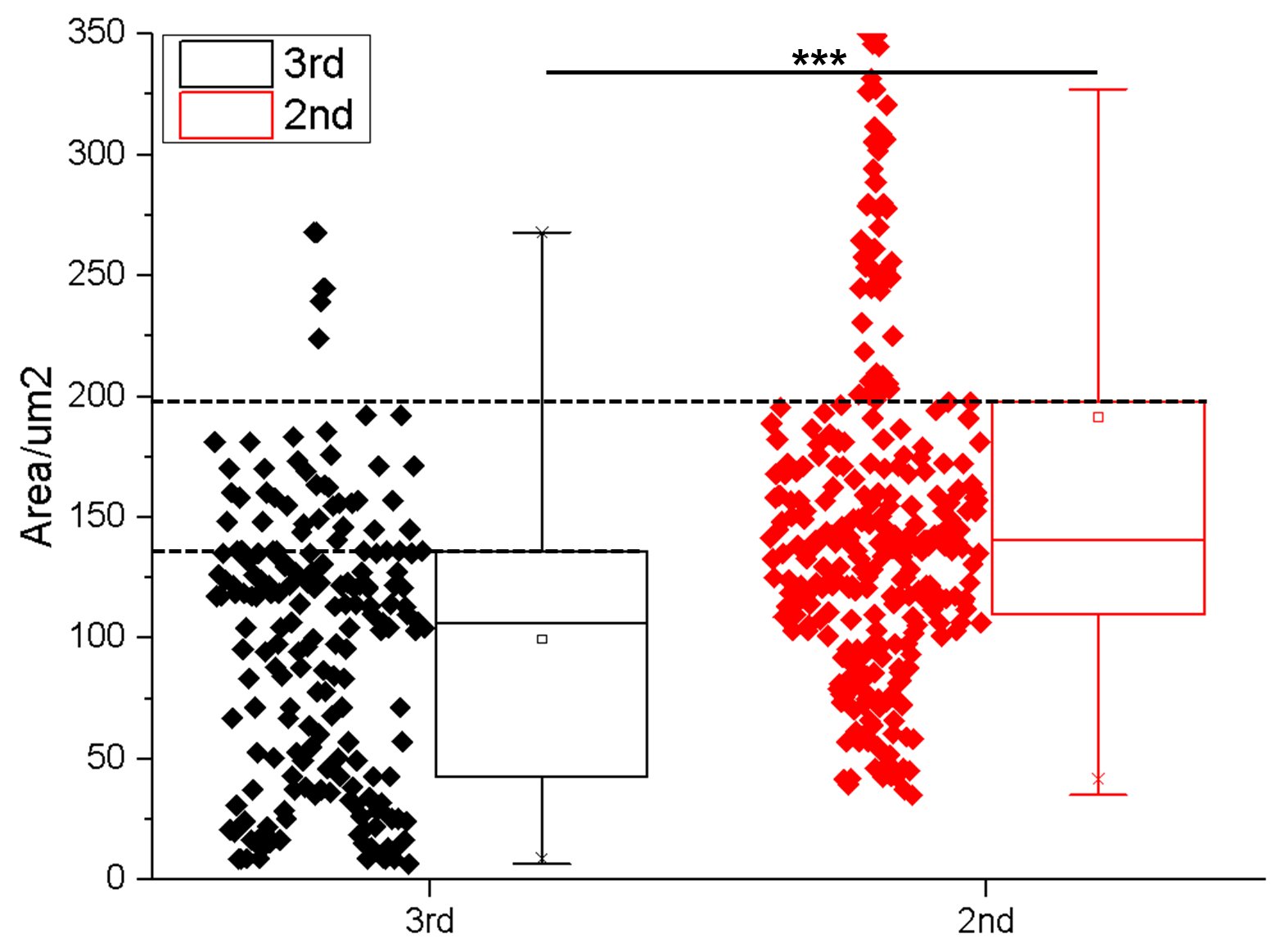


**Supplementary Figure 1: Box plot demonstrating the size separation thresholds obtained with the curved micro-channels for the middle and outer outlets respectively.** Dotted lines indicated the estimated thresholds for respective outlets. P-value is < 0.00001**.**


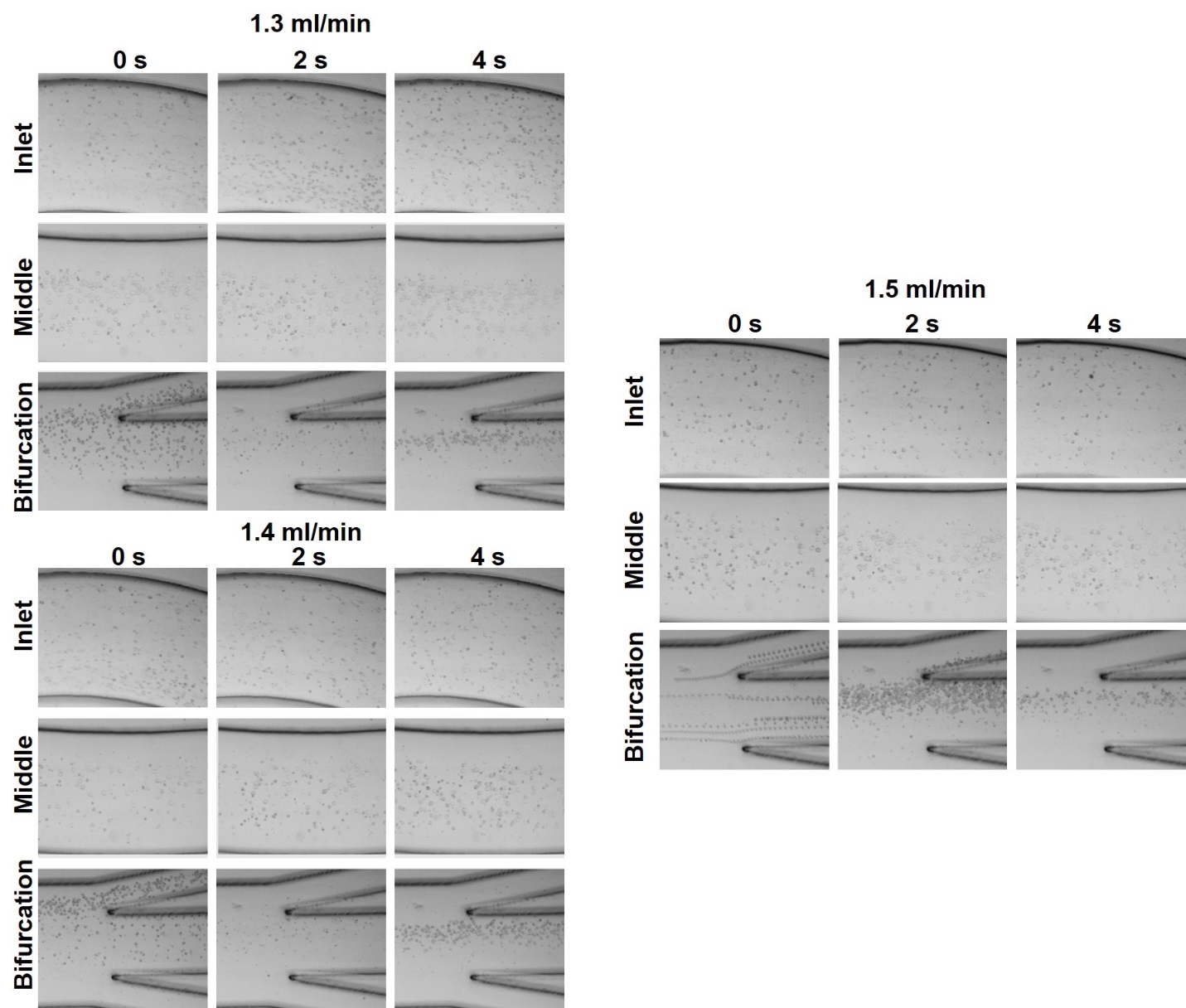


**Supplementary Figure 2. Flow profiles of cells within the BCB.** Characterization of target blast cell and WBC distribution under various flow rates


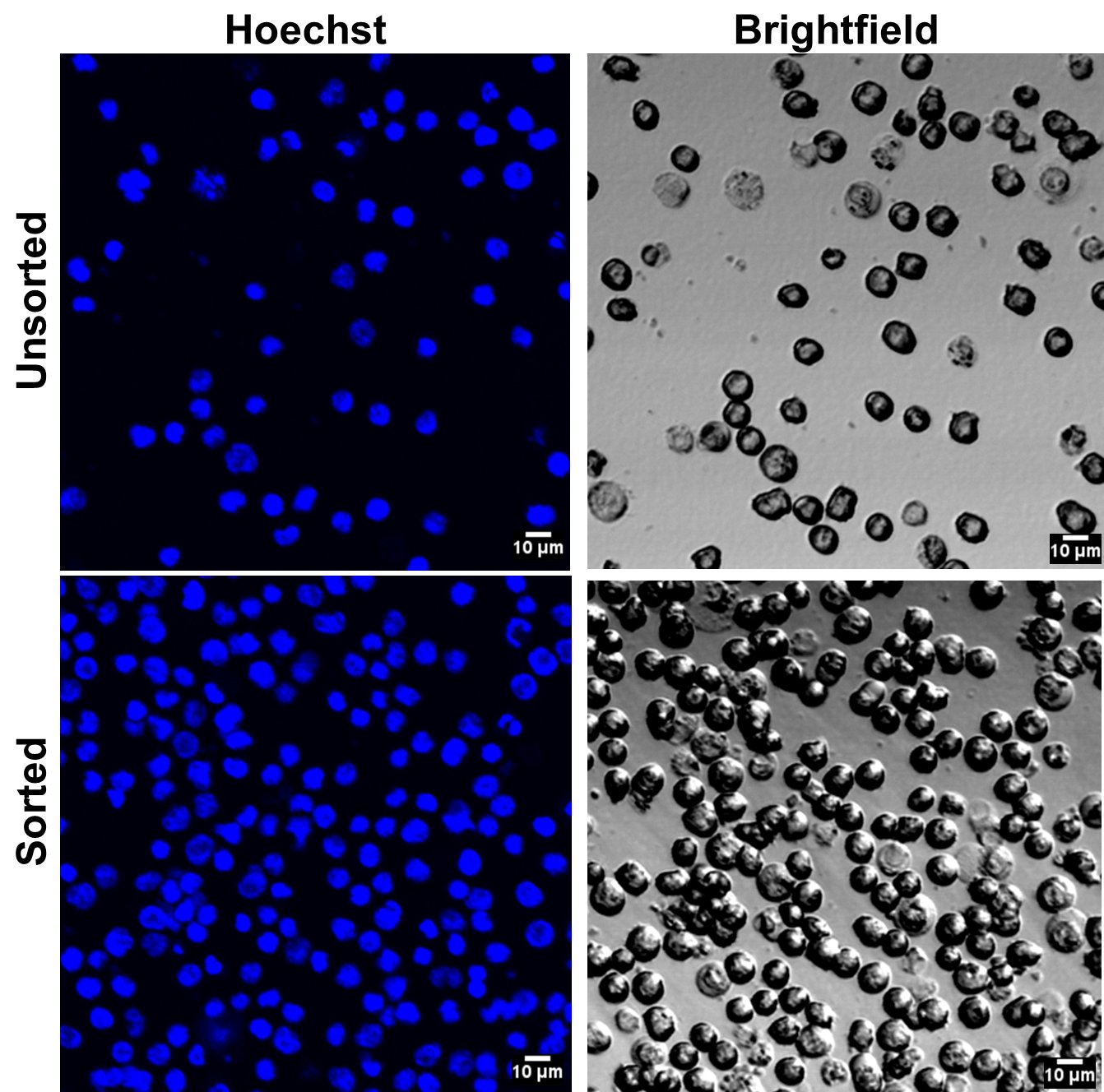
 **Supplementary Figure 3. Visualisation of blast cells before and after device sorting procedures.** Sorted cell samples did not display distinct alterations to morphology. Scale bar is 10 μm.

**
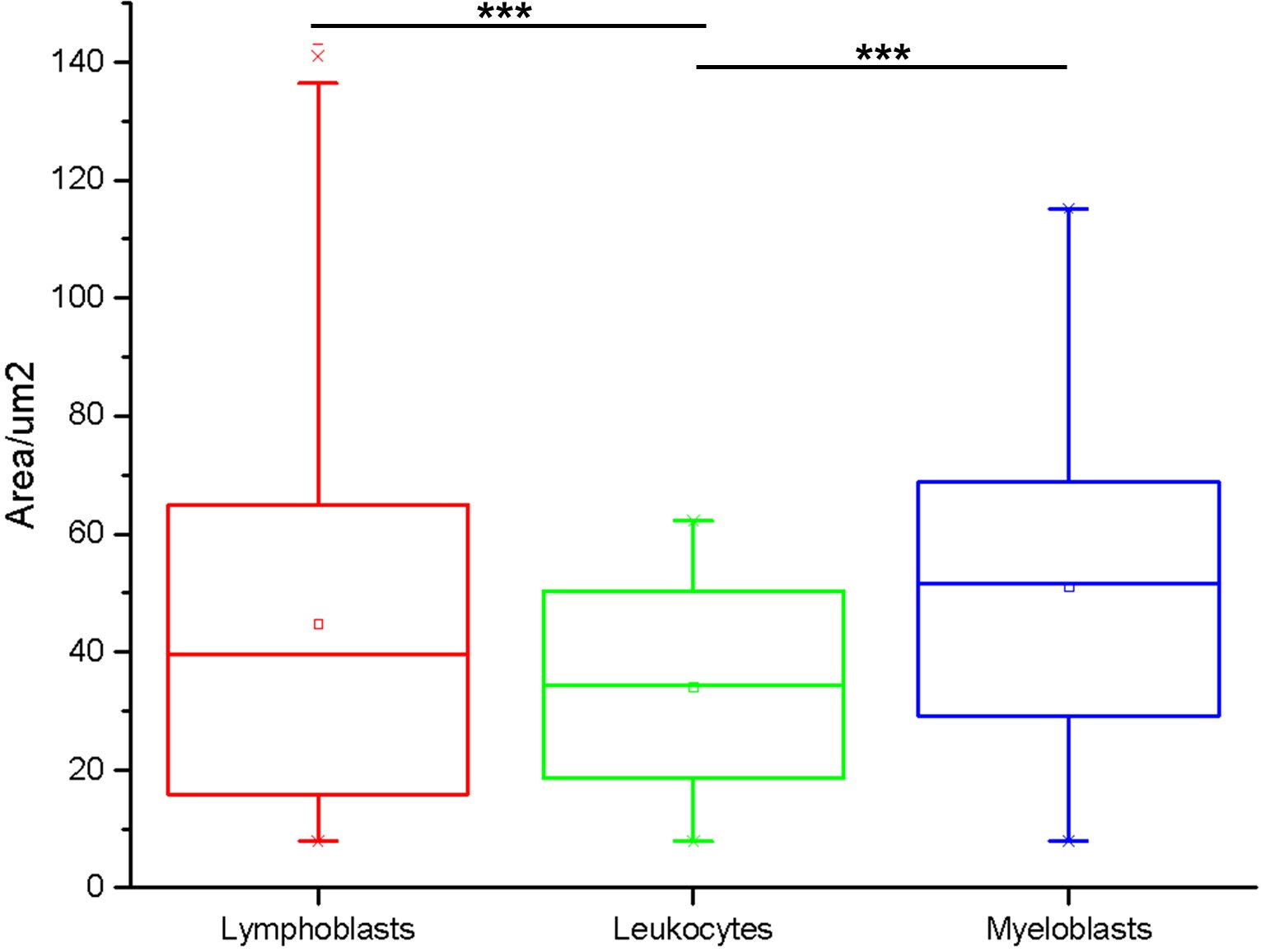
Supplementary Figure 4. Box plot demonstrating the range of cell size by area.** The cell size range of the blast cell types (lymphoblasts and myeloblasts) was similar but significantly different from leukocytes. P-value is < 0.00001.


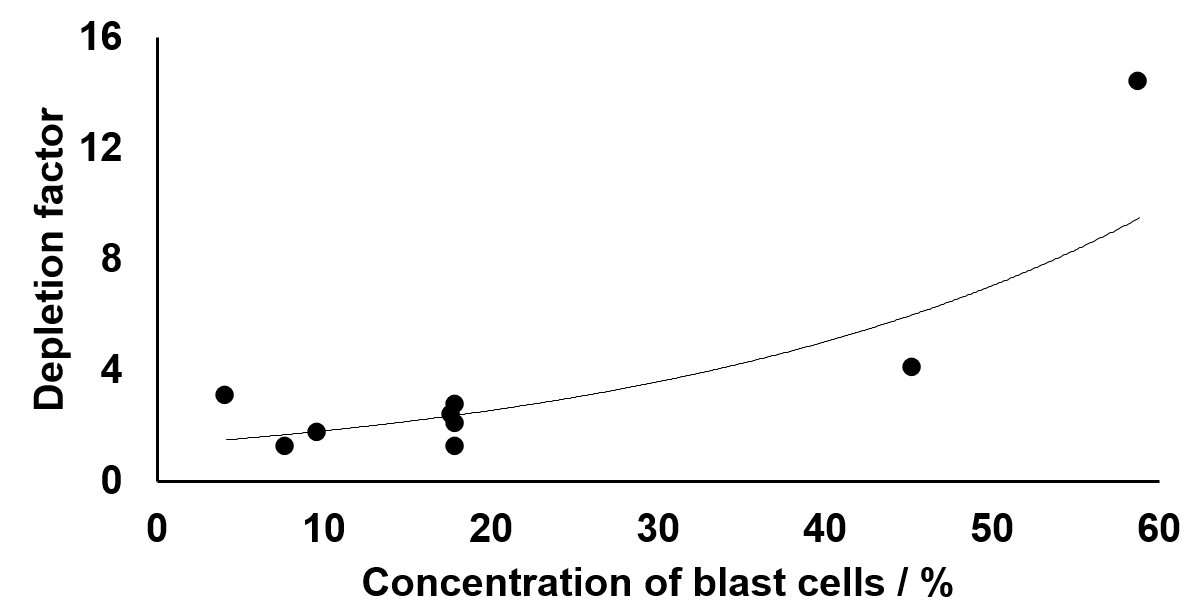


**Supplementary Figure 5. Scatter plot demonstrating the depletion factor of leukocytes in actual clinical samples in a single run.** Leukocyte counts could be reduced by a range of 1.28-14.4 folds within a single run.


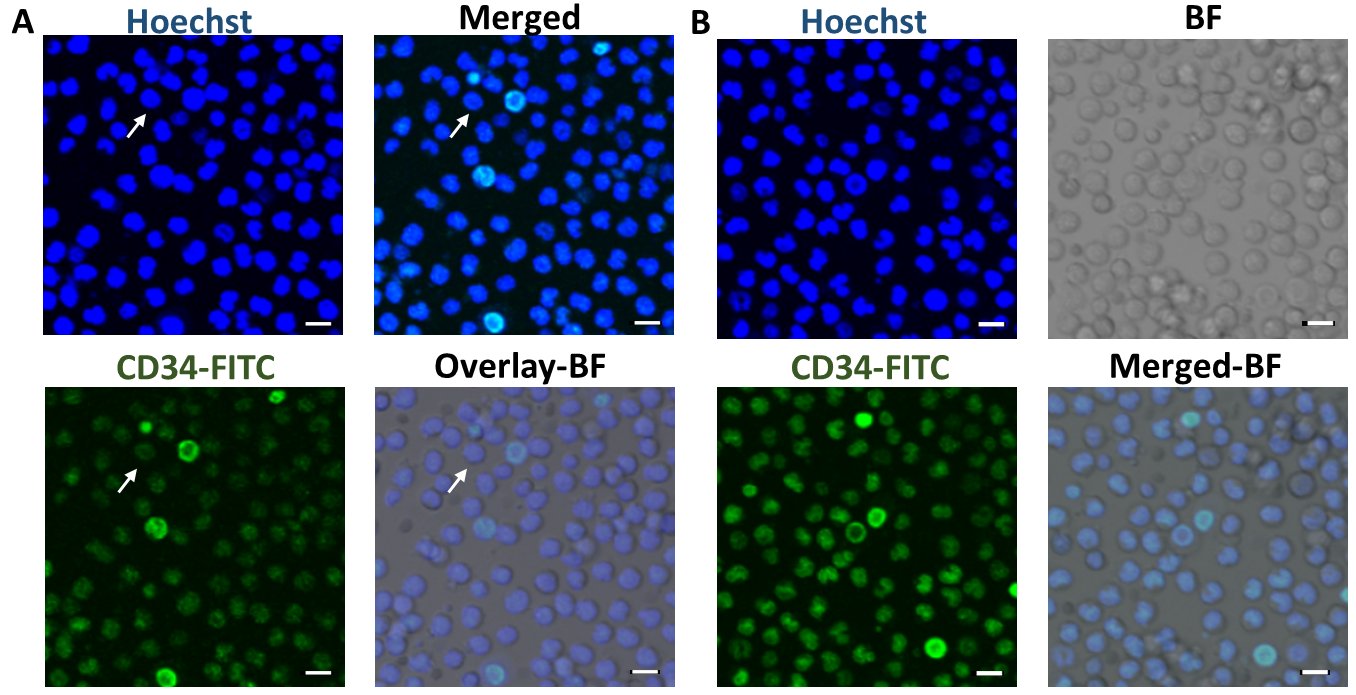


**Supplementary Figure 6. Other leukemia cells recovered from the BCB. (**A) CD34- cells with high N/C ratio. (B) Megakaryocyte-like large cells. Scale bar is 20 μm.

**Supplementary Table**

**Supplementary Table 1: Patient demographics of sample cohort.** Period of relapse or survival is indicated as the number of months from the date of sample collection. ID: patient identification code; NA: not available; CR: complete response; N: no; Y: yes; CVAD: Cyclophosphamide, Vincristine, Adriamycin, and Dexamethasone; POMP: 6‐mercaptopurine (Purinethol), vincristine (Oncovin), methotrexate, and prednisone; FLAG-IDA: Fludarabine, Idarubicin and High Dose Cytarabine. MUD: Matched unrelated donor; MSD: Matched sibling donor; MRD: matched related donor; and HiDAC: High-Dose Cytarabine.

| ID | Age | Gender | Treatment | Response | Relapse | Survival |
| --- | --- | --- | --- | --- | --- | --- |
| P-J | 43 | M | R-HyperCVAD + POMP Maintenance | CR | Y (16) | N (19) |
| GLL | 75 | M | Best Supportive Care (transfusion) | NA | NA | N (8) |
| LSK | 63 | F | FLAG+IDA - MUD transplant | CR | N | Y |
| ABB | 56 | F | FLAG+IDA | NA | NA | N (1) |
| MAS | 77 | M | NA | NA | NA | N.A. |
| D-D | 70 | M | Catarabine + Daunorubicin + Quizartinib/placebo (QUANTUM-First Trial) +Haplo transplant | CR | N | Y |
| NTM | 74 | F | Palliative treatment - Azacytadine - phase 1 trial (Midostaurin+Panobinostat) - SC Cytarabine | NA | NA | N (3) |
| DSP | 51 | F | FLAG-IDA + Azacitidine consolidation + MSD transplant | CR | N | Y |
| GJDDC | 54 | M | NA | NA | NA | N.A. |
| GKL | 55 | M | Catarabine + Daunorubicin + Quizartinib/placebo (QUANTUM-First Trial) + MSD transplant | CR | NA | Y |
| WYC | 39 | F | Cytarabine/Daunorubicin (3+7 Induction) + HiDAC (Consolidation) + MRD transplant | CR | NA | Y |
| KBH | 62 | F | Cytarabine/Daunorubicin (3+7 Induction) + HiDAC (Consolidation)  - FLAG-Ida - Best supportive care (Azacitidine + Venetoclax) | Refractory Disease | NA | N (13) |
| MTSB | 49 | F | Induction therapy (3 + 7), Azacitidine + Sorafenib, Ara C 3g/m2 + Sorafenib, Ara C 3g/m2 + Sorafenib, Sorafenib Maintainance, Azacitidine Maintainance before transplant, Cord blood transplant | CR | NA | Y |
| CPF | 73 | M | Sorafenib and Azacitidine | CR | Y (12) | N (16) |
| TKH | 51 | M | Cytarabine/Daunorubicin (3+7 Induction) + HiDAC (Consolidation) + MUD transplant | CR | N | Y |
